# The Story of CD4^+^CD28^−^ T Cells Revisited: Solved or Still Ongoing?

**DOI:** 10.1155/2015/348746

**Published:** 2015-03-05

**Authors:** Kathrin Maly, Michael Schirmer

**Affiliations:** Clinic VI, Laboratory of Molecular Biology and Rheumatology, Medical University of Innsbruck, Anichstrasse 35, 6020 Innsbruck, Austria

## Abstract

CD4^+^CD28^−^ T cells are a unique type of proinflammatory T cells characterised by blockade of costimulatory CD28 receptor expression at the transcriptional level, which is still reversible by IL-12. In healthy individuals older than 65 years, these cells may accumulate to up to 50% of total CD4^+^ T lymphocytes as in many immune-mediated diseases, immunodeficiency, and specific infectious diseases. Here we focus on CD4^+^CD28^−^ T cells in chronic immune-mediated diseases, summarizing various phenotypic and functional characteristics, which vary depending on the underlying disease, disease activity, and concurrent treatment. CD4^+^CD28^−^ T cells present as effector/memory cells with increased replicative history and oligoclonality but reduced apoptosis. As an alternative costimulatory signal instead of CD28, not only natural killer cell receptors and Toll-like receptors, but also CD47, CTLA-4, OX40, and 4-1BB have to be considered. The proinflammatory and cytotoxic capacities of these cells indicate an involvement in progression and maintenance of chronic immune-mediated disease. So far it has been shown that treatment with TNF-*α* blockers, abatacept, statins, and polyclonal antilymphocyte globulins (ATG) mediates reduction of the CD4^+^CD28^−^ T cell level. The clinical relevance of targeting CD4^+^CD28^−^ T cells as a therapeutic option has not been examined so far.

## 1. Introduction

Nearly 20 years ago, a unique CD4^+^ T cell type lacking the expression of costimulatory CD28 surface receptors was first described in rheumatoid arthritis (RA) patients [1, 2]. T cell receptor (TCR) stimulation as primary and CD28 costimulation as secondary signal are necessary for T cell activation. CD28 is further involved in survival, IL-2 production, metabolic activity, and clonal expansion of T cells [1–3]. As these cells also lack CD28 mRNA [4], it became clear that CD28 expression is blocked at the transcriptional level. Downstream of the TATA box in the promoter region of the* CD28* gene, there are two regulatory motifs, called sites *α* and *β*, as described in two transformed T cell lines (CD28^+^ Jurkat and CD28^−^ HUT78), two primary cell lines (H28P and H28N), and two T cell clones (PL65 and K2) [5, 6]. These sequences represent the initiator element, which supports the strength of the promoter [7]. The CD28 *αβ*-initiator is activated by the binding of a protein complex consisting of the site *α* specific proteins nucleolin and the A isoform of heterogeneous nuclear ribonucleoprotein-D0 (hnRNP-D0A). Both proteins were separately found in the CD28^+^ Jurkat and the CD28^−^ HUT78 T cell lines, but as a complex they were only detectable in CD28^+^ T cells, suggesting that complex formation is disturbed by posttranscriptional changes, such as phosphorylation of serine or threonine residues [6]. The exact mechanism causing the inhibition of the complex formation and further the loss of CD28 is still unclear. Nevertheless, the provided data support the hypothesis of a cell type distinguishable on transcriptional level, due to the lack of CD28 mRNA. A notable factor involved in CD28 downregulation is TNF-*α*, an inflammatory cytokine also produced in CD4^+^CD28^−^ T cells itself [8, 9], leading to downregulation of surface CD28 and affecting mRNA expression by long term exposure to Jurkat cells [10]. Also peripheral blood mononuclear cells cocultured with TNF-*α* showed downregulation of the CD28 costimulatory receptor [11], and high levels of TNF-*α* are associated with higher levels of CD4^+^CD28^−^ T cells in patients with unstable angina [12], supporting the findings of the in vitro studies. In contrast to TNF-*α*, exposure to IL-12 leads to reexpression of CD28 on CD4^+^CD28^−^ T cells isolated from peripheral blood mononuclear cells but has no effect on CD4^+^CD28^+^ T cells [3]. In these experiments, however, IL-12/TCR costimulation resulted in reexpression of CD28 in about 50% of the CD28^−^ T cells, suggesting heterogeneity in this cell population. The reexpressed CD28 receptors are functional, meaning the cells regain their complete costimulatory signalling capacity, including the following expression of responsive genes, like the CD40-ligand (CD40L). It is still a question to solve how IL-12 can reverse transcriptional inhibition of the CD28 gene by modifying the complex proteins nucleolin and hnRNP-D0A [3].

Taken together, loss of CD28 transcription is a typical feature of a specific CD4^+^ T cell subset but is still reversible in some cells. This review focuses on the characteristics of CD4^+^CD28^−^ T cells and their physiological and pathogenetical role in humans.

## 2. Prevalence, Oligoclonality, and Early Ageing of the CD4^**+**^CD28^−^ T Cell Subtype

### 2.1. Prevalence in Healthy Humans and Patients

Usually both CD4^+^CD28^−^ and CD8^+^CD28^−^ T cells are present in the peripheral blood, but CD4^+^CD28^−^ T cells occur less frequently than their CD8^+^ counterparts [13]. In healthy individuals older than 65 years CD4^+^CD28^−^ T cells accumulate to up to 50% of total CD4^+^ T lymphocytes [3, 5, 10], whereas healthy, young people have only few CD4^+^CD28^−^ T cells accounting for 0,1 to 2,5% of all CD4^+^ T cells [1, 14]. The enormous accumulation of CD4^+^CD28^−^ T cells with age indicates an involvement of the immune system in aging and represents CD28 as a marker of T cell aging [5].

At first, researchers detected elevated levels of CD4^+^CD28^−^ T cells in patients with RA [15, 16] followed by a broad range of other immune-mediated diseases, (like ankylosing spondylitis (AS) [9, 17], dermatomyositis and polymyositis [18], polymyalgia rheumatica and giant cell arteritis [19], systemic lupus erythematosus [20], granulomatous polyangiitis [21], multiple sclerosis [22], and active Crohn's disease [23]), in acute coronary syndrome [24–26], abdominal aortic aneurysms [27], and chronic kidney graft rejection [28] and even in infectious diseases, like infections with cytomegalovirus (CMV) [29, 30], human immunodeficiency virus (HIV) [31, 32],* Trypanosoma cruzi *[33], and chronic hepatitis B [34].

In summary, prevalence of CD4^+^CD28^−^ T cells is high in chronic inflammatory diseases [25, 35], immunodeficiency [28, 32], and specific infectious diseases [36]. Based on these findings, presence of CD4^+^CD28^−^ T cells has been proposed as a biomarker not only for normal aging, but also for early aging of the immune system under pathological conditions [5]. However, phenotypic and functional characteristics of CD4^+^CD28^−^ T cells may vary depending on the underlying disease, disease activity, and concurrent treatment and were not performed at the same extent in all of the mentioned diseases.

### 2.2. Oligoclonality

Interestingly, CD4^+^CD28^−^ T cells show limited TCR diversity [2] in the peripheral blood and form oligoclonal populations [1, 37], most likely as the result of repeated exposure to the same antigen. These clonally expanded CD4^+^CD28^−^ T cells were also found in the synovia of RA patients even though some of the detected clonotypes were CD28^+^, suggesting that the special environment of the synovia may support reexpression of CD28 on CD4^+^ T cells [1].

The permanent activation resulting in oligoclonality could be triggered by either endogenous or exogenous antigens including viral antigens expressed during chronic viral infections. One of the analysed viruses in this context, the human cytomegalovirus (CMV), which occurs in more than 90% of the elderly [38, 39], is still discussed as a candidate for ongoing stimulation of the immune system. However, the high infection rate of CMV results in a virtually absolute cooccurrence with CD4^+^CD28^−^ T cells. A good argument for CMV involvement is the finding that 30-fold more CD4^+^CD28^−^ T cells respond to CMV compared to their CD28^+^ counterparts [30]. CMV alters production of diverse cytokines in host cells possibly supporting the induction of the CD4^+^CD28^−^ T cell phenotype. For example, in dendritic cells CMV infection may lead to cellular activation via the viral Toll-like receptors (TLR) 7 and 9 and secretion of high amounts of IFN-*α*, causing downregulation of the costimulatory molecule CD28 [40, 41]. Proliferation and cytokine production of CD4^+^CD28^−^ T cells are induced by CMV stimulation but not by stimulation with other antigens like tetanus toxin or varicella-zoster virus. Viral DNA of CMV was also found in the synovial tissue and fluid of RA patients and is associated with the development of cardiovascular diseases, suggesting a common pathogenetic background of these diseases with CMV and CD28^−^ T cell expansion [29, 30]. This hypothesis is supported by a study, which shows that CD4^+^CD28^−^CD57^+^ T cells were only expanded in CMV seropositive RA patients, indicating a previous CMV infection [42]. Moreover, some AS patients were also tested positive for CMV and Epstein-Barr virus (EBV), but only titres of immunoglobulin (Ig) G for CMV but not for EBV correlated with prevalence of CD4^+^CD28^−^ T cells [17]. These results together with the finding that CD4^+^CD28^−^ T cells could be stimulated by CMV antigens strongly suggest an involvement of CMV in CD4^+^CD28^−^ T cell activation. Besides, it was shown that more than 50% of cultured CD4^+^CD28^−^ T cells from patients suffering from acute coronary syndrome recognize the 60 kD human heat-shock protein (hHSP-60), an ubiquitously expressed intracellular chaperone protein. However, hHSP-60 could only be recognized by CD4^+^CD28^−^ T cells from coronary artery disease patients during the acute phase and not from patients with chronic stable angina during the stable phase or healthy individuals [43]. Heat-shock proteins can be expressed by all cells under specific stress conditions, including increased temperature, an inflammatory environment or oxidative stress [44]. The detection of HSP-reactive T cells suggests that these proteins represent self-antigens triggering immunoregulatory pathways in inflammatory diseases [44]. The production of IFN-*γ* and perforin after activation of CD4^+^CD28^−^ T cells with hHSP-60 confirms its stimulatory feature and indicate, especially due to its ubiquitous expression that hHSP-60 is the factor responsible for permanent T cell activation leading to oligoclonality in acute coronary syndromes [43]. hHSP-60-TCR interaction alone could not induce the cytotoxic phenotype of these special T cells in contrast to the interaction with KIR2DS2, suggesting a T cell activation via NK receptors [45]. At the current state there are only data available regarding the role of hHSP-60 in activation of CD4^+^CD28^−^ T cells in acute coronary syndrome patients. It would be of interest if this interaction, hHSP-60-KIR2DS2, is also observable in other diseases with KIR2DS2 expressing CD4^+^CD28^−^ T cells [46].

### 2.3. Increased Replicative History and Reduced Apoptosis

The telomere length becomes shorter after each cell division, and at the point, which is also known as the “Hayflick limit” when the telomeres reach a critically short length, the cells are senescent and undergo apoptosis [47]. The CD4^+^CD28^−^ T cells found during ageing and in immune-mediated diseases also show significantly shortened telomeres indicating a replicative history [36, 48, 49]. Normally, telomerases are the enzyme complexes responsible for prolongation of shortened telomeres [50]. As telomerase activity depends on the expression of CD28, it decreases with CD28 reduction and could explain the shortened telomere length in CD4^+^CD28^−^ T cells [48]. This phenomena have been studied in aged CD4^+^ T cells both in RA and axial spondyloarthritis (including AS) so far [51, 52]. One could expect that loss of the most important costimulatory signal CD28 and a shortened telomere length result in anergy and apoptosis [5, 53]. However, CD4^+^CD28^−^ T cells show the opposite; they undergo less apoptosis than their CD28^+^ counterparts [16, 54] and are able to survive in the periphery over years [36, 55]. One cause of apoptosis is activation-induced cell death (AICD), eradicating activated T cells by generation of a death signal after Fas-FasL interaction, leading to phosphorylation of the Fas receptor death domain and finally to cell death by activation of several cascades, including caspases 1, 3, 8, and 10 [56, 57]. The inhibitor of this pathway, the Fas-associated death domain-like IL-1-converting enzyme inhibitory protein (FLIP), however, is increased in CD4^+^CD28^−^ T cells and interacts with caspases 8 and 10 or with the Fas-associated death domain and thus inhibits cell death by interrupting Fas signalling [55]. Besides, analysis of bcl-2 family members revealed an antiapoptotic effect with increased bcl-2 levels in CD4^+^CD28^−^ T cells after withdrawal of IL-2 as apoptotic stimulus [16].

## 3. Characteristics of CD4^+^CD28^−^ T Cells

### 3.1. Functional Aspects and Origin

**Figure 1:**
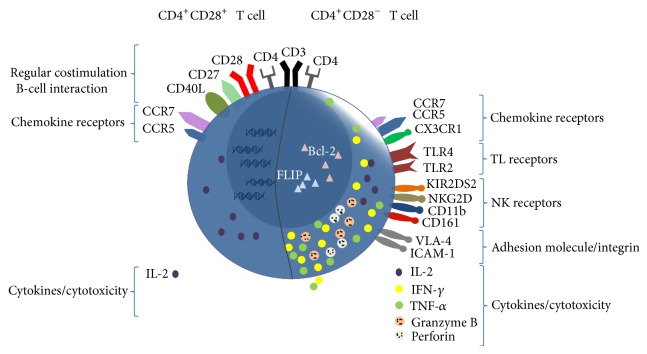
*Phenotypic and functional features of CD4* ^*+*^ *CD28* ^*−*^ *T cells and their CD28* ^*+*^ *counterparts (overview)*. Schematic representation of CD4^+^CD28^+^ (left half) and CD4^+^CD28^−^ (right half) T cells characterized by their specific surface marker and cytokines. Brackets on the sides describe the function, origin, or property of the receptors or cytokines. The size and the number of the displayed elements define their expression level.

As CD4^+^CD28^−^ T cells produce large amounts of IFN-*γ*, IL-2, so as the cytotoxic molecules perforin and granzyme B, they can be classified as cytotoxic T-helper 1-type cells [46, 54, 58, 59] (see Figure 1). Usually, Th1 cells are characterized by the secretion of cytokines such as IFN-*γ* and mediate in this way inflammatory reactions but have no cytotoxic features [60]. Granzyme B and perforin are expressed virtually in nearly all CD4^+^CD28^−^ T cells. Activation of these cells results in a reduction of intracellular granzyme B and perforin levels, suggesting that activation leads to degranulation, release, and further cytotoxicity [61]. In vitro studies showed that the cytotoxicity of these cells causes cell death of endothelial cells [62]. The secretion of the proinflammatory cytokines TNF-*α* and IFN-*γ* promotes the inflammatory environment whereas local TNF-*α* contributes to the downregulation of CD28 [63]. Particularly the proinflammatory cytokine IFN-*γ* is highly produced and involved in the development of rheumatoid synovitis but also in atherosclerosis development [64]. It is suggested that the secretion of IFN-*γ* mediates disease by causing the perturbation of inflammation. As a possible consequence of this Th1 profile, activation of macrophages by IFN-*γ* leads to secretion of matrix metalloproteinases, which are implicated in tissue damaging processes [65]. The cytotoxic properties of granzyme B and perforin may result in destabilization of unstable plaques and culprit lesions [62] as well as damage of the vascular smooth muscle cells and endothelial layer by these cells in vitro, amplified by high sensitivity-C-reactive protein [62, 66]. The C-reactive protein has direct proinflammatory effects on T cells [67] and is also able to increase the sensitivity of HUVECs (human umbilical vein endothelial cells) to the cytotoxicity of these special T cells in culture [62].

Increased production of IFN-*γ* and perforin by CD4^+^CD28^−^ T cells has been shown not only in RA but also in AS [9, 17], polymyalgia rheumatica/giant cell arteritis [15, 19], granulomatous polyangiitis [21], and abdominal aortic aneurysms [27]. Classification of CD4^+^CD28^−^ as Th1-type T cells has also been supported by the chemokine receptor expression profile, which is similar to that of Th1 cells, meaning upregulation of CXCR3 as well as the CXCR3/CCR4 ratio as identified in patients with AS. Particularly expression of Th1 receptor CXCR3 strongly indicates that these T cells derive from CD28^+^ Th1 cells [9]. To further examine the relationship between IFN-*γ* producing CD4^+^CD28^−^ T cells and Th1 cells the DNA methylation status of the IFNG locus was analysed. Hypomethylation of the IFNG locus is induced during the differentiation to Th1 cells and leading further to the expression of IFN-*γ*. This hypomethylation of the IFNG locus was also detected in CD4^+^CD28^−^ T cells. The similarity of their DNA methylation patterns further supports the Th1-type of CD4^+^CD28^−^ T cells [68].

### 3.2. Phenotypic Characteristics

Beside the loss of CD28 these proinflammatory CD4^+^ T cells show even more typical phenotypes different from their CD28^+^ counterpart, at least in part with the potential of alternative costimulation of the CD28^−^ T cells (see Figure 1). For example, expressions of CD7 [1] and CD40L [58, 69] are reduced and the receptor CX3CR1 [63], the natural killer (NK) cell receptors NKG2D [19, 70], CD11b [71], CD57 [71], KIR2DS2 [46], CD161 [72], and Toll-like receptor (TLR) 2 and TLR4 upregulated on CD4^+^CD28^−^ T cells [73].

#### 3.2.1. Adhesion Molecules and B-Cell Interaction Using the CD40-Ligand

ICAM-1 is a surface adhesion molecule densely expressed on CD4^+^CD28^−^ T cells in RA and multiple sclerosis (MS) patients, assuming a high migratory potential. Cytotoxic CD4^+^CD28^−^ T cells in MS patients additionally express the VLA-4 and LFA-1 receptor, whereby the LFA-1 interaction with ICAM-1 promotes the secretion of proinflammatory cytokines by inducing various transmembrane signals [22, 74]. Interaction and communication of classical CD4^+^CD28^+^ T cells with B cells require CD40L (=CD154), which is important by binding CD40 on the B cell surface. Reduction of CD28 on CD4^+^CD28^−^ T cells coincides with a decreased expression of CD40L [58, 69], resulting in deficiency of the CD40-CD40L interaction. In vitro, four different CD4^+^CD28^−^ T cell clones failed to provide signals obligatory for B-cell differentiation and immunoglobulin secretion [58]. Furthermore, it has to be studied if this downregulation also influences B cell activation, recruitment of germinal centre precursors, and induction of memory B cells as it was observed in studies with normal CD4^+^ T cells [75]. Contrary to these findings a newer study showed an increase of CD40L in CD4^+^CD28^−^ T cells in bronchiolitis obliterans syndrome [76]. Future studies will address the question if expression of CD40L is unique for CD4^+^CD28^−^ T cells in bronchiolitis obliterans syndrome or not.

#### 3.2.2. Chemokine Receptors

Amongst the chemokine receptors, CX3CR1 on the surface of CD4^+^CD28^−^ T cells is best characterised in the peripheral blood of RA, MS, and primary sclerosing cholangitis patients [63]. These studies showed CD4^+^CD28^−^CX3CR1^+^ T cells in the synovial fluid of RA and in the brain of MS patients [74]. More than 60% of CD4^+^CD28^−^ T cells in RA patients expressed the CX3CR1 receptor, while their CD28^+^ counterparts were constantly negative for CX3CR1 [77]. This receptor interacts with the chemokine fractalkine, which is presented on the surface of cultured fibroblast-like synoviocytes in the synovial tissue from RA patients [77] as well as increasing in the serum of RA [78] and in the cerebrospinal fluid (CSF) of MS patients [74] compared to healthy controls. Furthermore, fibroblast-like synoviocytes stimulated with either TNF-*α*, IFN-*γ*, or both could induce the fractalkine mRNA expression in vitro [77], suggesting a similar role in vivo. Binding of fractalkine to CX3CR1^+^CD28^−^ T cells results again in enhanced inflammation with tissue damage after secretion of IFN-*γ* and TNF-*α* by the effector cells as well as angiogenesis [74, 79], supporting its pathogenic effects in patients with RA. In MS patients it was observed that the chemokine gradient of soluble fractalkine is responsible for migration of these cells to the inflamed brain lesions [74].

In RA, the expressed chemokine receptors include CXCR4 and CCR5, which are responsible for tissue invasion, and CCR7, the lymphoid homing chemokine receptor. Expression of the CCR5 and CCR7 receptors gives the T cells the abilities of short-lived effector and long-lived memory T cells by allowing them either to home into the lymphoid organs or to invade into tissues like the synovial fluid and membrane. Invasion into tissues and production of inflammatory cytokines then lead to an enhancement of local inflammation and autoreactivity [72, 80–82].

In contrast, CCR5 expression was extremely low in AS patients not only in CD28^−^ but also in CD28^+^ T cells, while CXCR3 was highly expressed in CD4^+^CD28^−^ T cells [9]. These data may explain why CD4^+^CD28^−^ T cells in AS have a reduced capacity to invade into synovial tissues compared to their CCR5-rich counterparts in RA.

#### 3.2.3. Natural Killer Cell Receptors

CD4^+^CD28^−^ T cells produce natural killer cell receptors, like CD11b and CD57 but lack their specific CD16 molecule. Despite NK receptor expression they are not classified as typical natural killer T cells due to the fact that their characteristic expression of an invariant TCR *α*-chain could not be detected [71]. In diseases like RA, granulomatous polyangiitis, Sjögren's syndrome, and even in insulin-dependent diabetes mellitus CD4^+^CD28^−^ T cells were detected, which are able to express NKG2D receptors [70] together with other killer cell immunoregulatory inhibitory receptors (KIR), mediating cellular cytotoxicity [71, 72, 83]. NKG2D is usually expressed on NK cells, CD8^+^ T cells but not on CD4^+^ T cells and interacts with its stress-inducible, major histocompatibility complex class I-chain related ligand (MIC) A and MICB, which are present in the intestinal epithelium [84] and the synovia of RA patients [83]. The activation by MIC causes survival of the autoreactive CD4^+^CD28^−^ T cells and leads to endocytosis and degradation of NKG2D [69, 85]. Patients suffering from RA have remarkable high levels of TNF-*α* and IL-15. This supports upregulation of NKG2D, reversing its downmodulation by MIC [70] and promoting the proinflammatory status as the tissue damaging effect [83]. Furthermore, NKG2D was also expressed on CD4^+^CD28^−^ T cells in giant cell arthritis and polymyalgia rheumatica, accumulating around the vasa vasorum in the temporal arteries suggesting a ligand interaction in this region [19].

The functional role and mechanism of KIRs are still elusive but a subtype of this family, KIR2DS2, was detected on the surface of CD4^+^CD28^−^ T cells from RA and acute coronary syndrome patients. It was confirmed that this receptor has active costimulatory functions and is considered to be a risk factor for the development of vasculitis but not synovitis. KIR2DS2s expressed on CD4^+^CD28^−^ T cells are supposed to recognize HLA-C molecules [46, 86] and human HSP-60 on major histocompatibility complex class 1 molecules, inducing the expression of perforin. Additionally, it was shown that cytokine expression of IFN-*γ* was independent of KIR2DS2 indicating separate pathways for proinflammatory and cytotoxic functions [43, 45, 87]. Whether expression of NK receptor CD161 on CD4^+^CD28^−^ T cells in RA patients is relevant remains open. The ligand of CD161 is not known, but it is suggested that it differs from KIR and NKG2D receptors. It is most likely that CD161 is involved in tissue invasion and in T cell adhesion [72].

#### 3.2.4. Toll-Like Receptors

Interestingly, not only NK receptors but also TLRs are expressed on CD4^+^CD28^−^ T cells [73]. TLRs represent one of the four classes of the pattern recognition receptor families [88, 89], which are expressed on cells of the innate immune system. Each of the 10 human TLRs recognizes specific pathogen-associated molecular patterns of either bacterial or viral origin [88, 90, 91]. For patients with RA, AS, and psoriatic arthritis (PsA) it was confirmed that TLRs, sensitive to lipopolysaccharides (LPS), are expressed on CD4^+^CD28^−^ T cells. TLR4 was highly expressed on T cells in all three disease groups, while TLR2 was only detectable on cells from AS patients, but in a lower frequency. Activation via LPS/TLRs increased the cytotoxic capacity of CD28^−^ T cells, which was shown by the upregulated intracellular release of perforin [73]. The induction of the perforin expression resulted from a TCR independent activation of TLR4 on CD4^+^CD28^−^ T cells without a concomitant TCR cross-linking. The high affinity of TLR4 on CD4^+^CD28^−^ T cells to LPS indicates a possible involvement of components of Gram-negative bacteria, especially in AS [73].

### 3.3. Alternative CD28-Independent Costimulatory Pathways of CD4^+^CD28^−^ T Cells

CD28 costimulation is characterized amongst others by an upregulation of the IL-2 production and thus preventing efficiently apoptosis [80, 92]. Searching for an appropriate costimulatory pathway instead of CD28 with these same effects led to several different pathways as independent stimulators. Here we discuss the most important ones, but it can be expected that even more pathways can be involved to achieve production of IL-2 and prevent apoptosis without the CD28-CD80/86 pathway.

#### 3.3.1. CD47-Thrombospondin-1 Interaction

As the first alternative pathway Vallejo et al. had examined CD47, also called integrin associated protein. CD47 is constitutively and highly expressed on the surface of peripheral T cells and bound by its ligand thrombospondin-1 (TSP1), an extracellular matrix protein, which is in turn bound on CD36. Analysis of the CD47-TSP1-CD36 pathway in CD4^+^CD28^−^ T cells from RA patients resulted in a comparable production of IL-2 and proliferation rates as observed for the CD28 pathway. TSP1 occurs mostly on damaged and repairing tissues raising the question whether this costimulus is involved in T cell recruitment and tissue inflammation: indeed TSP1 is highly expressed in the synovia of RA patients and may support the migration of CD4^+^CD28^−^ T cells [80, 93, 94].

#### 3.3.2. Cytotoxic T-Lymphocyte-Associated Protein 4

An upregulation of cytotoxic T-lymphocyte-associated protein 4 (CTLA-4 = CD152) was detected on CD4^+^CD28^−^ T cells from healthy subjects [95] and patients with bronchiolitis obliterans syndrome [76]. CTLA-4 is a homolog of CD28 expressed on the surface of T cells, also activated by the CD28-ligands CD80 and CD86. CTLA-4 acts as immune downmodulator and inhibits IL-2 production as well as cell cycle progression. The antiapoptotic properties of the CTLA-4-CD80/86 pathway are mediated by disturbing the Fas-FasL interaction (see Section 2.3) through the inhibition of Bad. Furthermore, activation of CTLA-4 leads to an increase of Bcl-2 with its antiapoptotic effects [95].

#### 3.3.3. OX40 and 4-1BB

Both OX40 (*=CD134*) and 4-1BB (*=CD137*) are members of the tumour necrosis factor receptor family and are expressed on the surface of different cell types including activated T cells. They recognize their ligands OX40L and 4-1BBL [96] and use the tumour necrosis factor receptor associated factor adaptor molecules to activate PI-3 kinase, Akt/PKB, c-Jun N-terminal kinase, and the NF-*κ*B pathway [96, 97]. OX40 and 4-1BB are expressed and activated independent of CD28 on T cells and enhance cytokine secretion, proliferation, and survival (by upregulating bcl-2 family members) [96, 97]. CD4^+^CD28^−^ T cells detected in acute coronary syndrome patients express enhanced levels of OX40 and 4-1BB compared to their CD28^+^ counterparts. Inhibition of these costimuli resulted in decreased production of the proinflammatory cytokines IFN-*γ* and TNF-*α* and altered degranulation and release of perforin from CD4^+^CD28^−^ T cells. Despite the marginally changed intracellular level of granzyme B, blocking OX40 and 4-1BB could be a possible treatment option for acute coronary syndrome patients by inhibiting inflammation and cytotoxicity [61].

These and other important results helped to characterize CD4^+^CD28^−^ T cells with typical effector features, characterized by the expression of CD69 as indicator of activation, production of inflammatory cytokines, cytotoxic molecules, and expression of natural killer (NK) cell receptors, although it was found that they are still able to express the naïve T cell marker CD45RA [15, 27, 63, 98]. Because of their cytotoxic and proinflammatory features it is hypothesized that they might have an important role in the pathogenesis and maintenance of chronic immune-mediated diseases [35].

## 4. Aspects of CD4^+^CD28^−^ T Cells in Specific Disease

### 4.1. Recent Findings in Rheumatoid Arthritis and Ankylosing Spondylitis

The majority of the above-mentioned studies to characterize CD4^+^CD28^−^ T cells were performed with T cells from RA and AS patients. Therefore, this paragraph summarizes only some additional RA-specific information.

Interestingly, CD4^+^CD28^−^ T cells are found in one-third of all RA patients and occur with a higher frequency in the peripheral blood than in the synovial fluid [68, 99]. Recently, it was discovered that CD4^+^CD28^−^ T cells from the synovial fluid, but not from the peripheral blood were able to produce the proinflammatory cytokine IL-17. It is speculated that higher concentrations of TNF-*α* in the synovial fluid compared to the peripheral blood may induce additional cellular functions like production of IL-17 [68]. The amount of CD4^+^CD28^−^ T cells is directly associated with disease severity and the development of extra-articular manifestations with the highest levels of CD4^+^CD28^−^ T cells in patients with subcutaneous nodule formation and rheumatoid organ disease [2, 37, 100].

### 4.2. Role of CD4^+^CD28^−^ T Cells in Vascular and Vascular-Related Diseases

The main cause of acute coronary syndromes including unstable angina, myocardial infarction, and subsequent sudden cardiac death [26] is rupture of atherosclerotic plaques characterized by the inflammatory infiltration of macrophages and T cells [101]. CD4^+^CD28^−^ T cells occur frequently in patients with unstable angina but are rare in stable angina and healthy controls, leading to the speculation that these cells are involved in the development of cardiovascular events and apoptotic effects on vascular wall cells [24, 25]. The presence of this type of T cells in unstable but not in stable plaques indicates an important role in plaque disruption [26]. The high level of produced IFN-*γ* induces the recruitment and activation of macrophages and their secretion of metalloproteinases. The tissue damaging effect of metalloproteinases is well known and may result in the destabilization of the plaque, rupture, and superimposed thrombosis and thus raises the risk of acute ischemia [24, 62, 102]. CD4^+^CD28^−^ T cells in patients with acute coronary disease additionally produce enhanced levels of granulysin compared to CD28^+^ T cells, which is known to be involved in the immune defense against bacteria and fungi. This result together with the fact that myocardium infarction is more common after an infection strengthens the hypothesis that CD4^+^CD28^−^ T cells expressing TLRs might be activated by microbial agents [103]. For further confirmation it will be interesting if TLRs, as already found on the surface of CD4^+^CD28^−^ T cells in AS [73], are also present on cells in acute coronary disease and if cells from AS patients are also able to produce granulysin.

An increased risk for angina pectoris, myocardial infarction, and cardiac death has also been described for patients with rheumatoid vasculitis [64, 101] and patients with chronic kidney disease [104] depending on their level of CD4^+^CD28^−^ T cells. Also CD4^+^CD28^−^ T cells occur in diabetes mellitus [66], with higher levels in case of the first cardiovascular event and acute coronary syndrome compared to diabetes mellitus patients without a cardiovascular syndrome and patients with acute coronary syndrome but without diabetes mellitus. These cardiovascular complications could be in conjunction with the accumulation of glycosylated end-products and the poor glycaemic control, maybe associated with CD4^+^CD28^−^ T cells [66].

In patients with granulomatous polyangiitis CD4^+^CD28^−^ T cells were studied in the peripheral blood, the granulomatous lesions, and the bronchoalveolar lavage fluid. It was suggested that CD4^+^CD28^−^ T cells are recruited to granulomatous lesions by CD18-ICAM1 interaction and support in this manner granuloma formation by secreting inflammatory cytokines. However, it is not clear if these cells are recruited already in the CD28 negative status or still as CD28^+^ cells losing their receptor in the inflammatory environment of the granulomatous lesion after repeated activation [21, 98, 105].

### 4.3. Recent Findings in Primary Sclerosing Cholangitis and Bronchiolitis Obliterans Syndrome

Only recently the analysis of CD4^+^CD28^−^ T cells in patients with primary sclerosing cholangitis revealed that these cells occur with a frequency of 3,3% of all CD4^+^ T cells in the peripheral blood and 30,3% in the liver tissue, where they accumulate around the bile ducts. CX3CR1 was higher expressed on cells in the blood than in the liver tissue, but tissue infiltration was additionally promoted by CXCR6 and CCR10, which give the T cells the ability to migrate toward biliary epithelial cells. As a consequence the concentrations of TNF-*α* and IFN-*γ* increase with the accumulation of CD28^−^ T cells in the liver and induce in turn the expression of adhesion molecules and secretion of chemokines in biliary epithelial cells [63].

Bronchiolitis obliterans syndrome is also associated with increased levels of CD4^+^CD28^−^ T cells compared to stable lung transplant patients. Downregulation of CD28 was correlated with an increased need for steroids (steroid resistance) and an upregulation of specific costimulators OX40 (or CD134), 4-1BB (or CD137), CTLA-4 (CD152), and CD40L (CD154). The molecules 4-1BB and CTLA-4 are responsible for the production of cytotoxic T cells before or during graft rejection and OX40 and CD40L are important for proliferation and cytokine production of CD4^+^CD28^−^ T cells in bronchiolitis obliterans syndrome [76]. This finding is contrary to those of another study showing a downregulation of CD40L together with CD28 [58], indicating an alteration of CD4^+^CD28^−^ T cells in bronchiolitis obliterans syndrome. Indeed there is ongoing interest in the evaluation of the functional role of CD4^+^CD28^−^ T cells and their involvement in disease development, progression, or maintenance, and targeting CD4^+^CD28^−^ T cells as treatment option appears to be an intriguing topic for further studies.

## 5. Treatment-Induced Changes of CD4^+^CD28^−^ T Cells

Several studies concluded that the number of CD4^+^CD28^−^ T cells correlates with severity and extra-articular involvement in RA [82, 100]; additionally high levels of proinflammatory cytokine TNF-*α* were found in both the peripheral blood and synovia. Occurrence of CD4^+^CD28^−^ T cells together with TNF-*α* in RA patients raises the assumption that targeting TNF-*α* also reduce the level of cytotoxic T cells [10, 106]. Indeed, RA patients treated with disease modifying antirheumatic drugs like methotrexate showed no changes of cytotoxic T cells [28, 64, 100] but showed downregulation of CD4^+^CD28^−^ T cells and even reexpression of CD28 under treatment with the TNF-*α* inhibitor infliximab [11, 65]. Besides, TNF-*α* inhibition with infliximab led to downregulation of TLR expression on CD4^+^CD28^−^ T cells in AS patients [73]. However, CD4^+^CD28^−^ T cells could not be fully eradicated using infliximab, which results in a lowering of 33–36% of the CD28^−^ T cell level in RA and unstable angina patients [12, 64, 65].

Treatment of unstable angina patients with statins led to an even less pronounced reduction of CD4^+^CD28^−^ T cells [107]. Statins are 3-hydroxy-3-methylglutaryl-coenzyme A reductase inhibitors, resulted in reduction of soluble CD40L in patients with hypercholesterolemia [108] and coronary artery disease [109]. Independent trials to test the effect of statins on CD4^+^CD28^−^ T cells in patients with either stable coronary artery disease/unstable angina/acute coronary syndrome or diabetes mellitus show conflicting data with downregulation of CD4^+^CD28^−^ T cells in unstable angina and acute coronary syndrome, but lack of influence in the other two diseases [66, 103, 107, 109].

Reduction of CD4^+^CD28^−^ T cells was also observed in RA patients treated with abatacept [110]. Abatacept is a fusion protein consisting of the extracellular domain of CTLA-4 and the modified Fc portion of human IgG1. It binds the CD28/CTLA-4 ligands CD80 and CD86 and consequently inhibits their signalling pathway. Also the combination of abatacept with methotrexate showed a beneficial effect in RA patients, even after failure of other medications, like TNF-*α* blockers [110–112].

So far we are only aware of one approach for full eradication of CD4^+^CD28^+^ T cells, namely, that achieved by polyclonal antilymphocyte globulins (ATG) in vitro and in transplant patients [113]. ATGs are suppressors of the immune system used since decades as prophylaxis against graft versus host disease in transplant patients [114]. The high diversity of their antigens allows them to interact with all cell types of the peripheral blood mononuclear cell compartment and subsequently induces apoptosis in these cells. Analysis of T cells from transplant recipients treated with ATG revealed preferred eradication of CD4^+^CD28^−^ T cells in comparison with their CD28^+^ counterpart, suggesting a specific effect of ATGs against CD4^+^CD28^−^ T cells [113].

## 6. Conclusion

Ageing coincides with the development of CD4^+^CD28^−^ T cells showing typical phenotypes and costimulatory pathways. Thus properties from NK cells and monocytic lineages have been described as well as a functional role of these cells as Th1 cells. Granzyme B and perforin are involved in organ manifestations.

Many aspects of CD4^+^CD28^−^ T cells have been extensively described, although not in all diseases, but exemplary. To our understanding some important questions are still open: (1) why are not all cells restored by IL-12? (2) Can the observed phenomena be generalized for all diseases? (3) How to define the prognostic effect of CD4^+^CD28^−^ T cells, and could prognosis be improved by eradication of these cytotoxic cells? Finally we assume that the potential role of CD4^+^CD28^−^ T cells as biomarkers and prognostic factor is still underestimated for clinical practice, and it appears that there is still an ongoing strong interest of the scientific community in these cells.

## Abbreviations

AICD: Activation-induced cell death
AS: Ankylosing spondylitis
ATG: Antilymphocyte globulins
CSF: Cerebrospinal fluid
CTLA-4: Cytotoxic T-lymphocyte-associated protein 4 (=CD152)
CMV: Cytomegalovirus
EBV: Epstein-Barr virus
FLIP: Fas-associated death domain-like IL-1-converting enzyme inhibitory protein
hHSP-60: Human 60 kD heat-shock protein
HIV: Human immunodeficiency virus
hnRNP-D0A: Heterogeneous nuclear ribonucleoprotein-D0
HUVEC: Human umbilical vein endothelial cell
Ig: Immunoglobulin
LPS: Lipopolysaccharides
MS: Multiple sclerosis
NK cells: Natural killer cells
RA: Rheumatoid arthritis
TCR: T cell receptor
TLR: Toll-like receptor
TSP1: Thrombospondin-1.
