# Corrigendum to “The Story of CD4^**+**^CD28^−^ T Cells Revisited: Solved or Still Ongoing?”

**DOI:** 10.1155/2015/251657

**Published:** 2015-07-08

**Authors:** Kathrin Maly, Michael Schirmer

**Affiliations:** Clinic VI, Laboratory of Molecular Biology and Rheumatology, Medical University of Innsbruck, Anichstrasse 35, 6020 Innsbruck, Austria

In the article entitled “The Story of CD4^+^CD28^−^ T Cells Revisited: Solved or Still Ongoing?” [1], we wrote the following in Section 2.3. (Increased Replicative History and Reduced Apoptosis) about the telomere length and the “Hayflick limit”: “when the telomeres reach a critically short length, the cells are senescent and undergo apoptosis [47].” It has to be corrected as follows: “when the telomeres reach a critically short length, normal human cells become senescent [47].”
